# The Consequences of Tongue Piercing on Oral and Periodontal Tissues

**DOI:** 10.1155/2014/876510

**Published:** 2014-01-29

**Authors:** Ioannis Plastargias, Dimitra Sakellari

**Affiliations:** ^1^School of Dentistry, Aristotle University of Thessaloniki, GR-54124 Thessaloniki, Greece; ^2^School of Dental Medicine, University of Connecticut, Farmington, CT 06030, USA

## Abstract

This paper is discussing the potential consequences that may arise by the implementation of piercing in the oral cavity and is also categorizing the consequences according to their extent and severity. Furthermore, this paper is reviewing some possible oral hygiene methods that can prove to be auxiliary in decreasing the potential complications arising from oral piercing. This literature review is based on articles published from 1985 to 2012.

## 1. Introduction

Body piercing involves the puncturing of specific places of the body in which metallic adornments are installed. Piercing in the oral cavity has gained a rapid interest among the youth in the western world [1]. This interest may be attributed to several contributing factors. According to Kustner et al., the principal reason is the zest of the youth for being in style and in fashion. Other factors may include religion, traditional issues, rituals, or the feeling of being a member of a social group or even the feeling of superiority above the other members of the social “caste” [2]. As stated by Stirn et al., self-expression, expression of independence of spirit, amelioration of the body and of sensuality [3–5], and daring are contributing factors as well [6]. In their study it is also mentioned that oral piercing has been speculated to have healing results on depression. As a result it is postulated that the traumatic psychological events are correlative to piercing [3]. It has also been presented in studies that the practice of oral piercing is perceived as an aloof and bizarre behavioral pattern by society. This is a reason why the majority of patients who present to the dental office and use to wear piercings often take them off before the clinical session [7]. As a result, edema of oral soft tissues may be attributed to preexisting oral piercing that cannot be seen by the dentist though [8].

The overall purpose of this paper is to review the potential complications caused by oral piercings as they are analyzed in the literature. This paper also suggests some ways of improving the oral hygiene of the people who wear piercings and it suggests some methods of ameliorating the negative consequences of piercings.

## 2. General Literature Data on Oral Piercings

*There are some popular oral piercing spots*: it is a general rule that the most common form of piercing is the barbell type piercing and the mostly pierced oral site is the tongue [9]. It has been reported that the tongue is often pierced in the midline and more specifically in the median lingual sulcus, albeit some piercings are performed on the dorsolateral site of the tongue anterior to the lingual frenum [10]. Other reports in the literature suggest that only 45% out of 108 patients were pierced at their tongue and that only 5% of these patients combined a tongue piercing and a labret piercing. Other reports also suggest that the labiomental groove is another popular spot of piercing [11]. The anterior side of the tongue is also reported as a piercing spot.

*Oral piercings come in some popular shapes*: regarding the shape of the piercings, the most common shape of the tip of the piercing presented is the ball-shaped one (94% of the cases) with cone-shaped being the next (4% of the cases) and cylindrical being the least popular, with only 2% of the presented cases. The material used for the fabrication of piercings is titanium (65%), steel (25%), acrylic (6,3%) and niobium (5%) [12].

*It is also crucial to examine the sociological viewpoint of the pierces*: from a sociological point of view, most of the participants had middle school level of education (46% compared to 37% of the population) and elementary school (50%), most of them were smokers, they were frequent consumers of alcohol, and they applied satisfactory oral hygiene by brushing their teeth two times a day [12].

## 3. Perioperative and Postoperative Consequences 

In a case series report a young female patient was described who had her tongue pierced and showed gingival recessions with no symptoms at the lingual surface of the mandibular central incisors. She presented with reasonable oral hygiene and probing depths, whilst her gingiva were erythematous (moving to the alveolar mucosa) with partly white appearance at the gingival margin [13]. The symptoms described above imply the negative repercussions of tongue piercing, including gingival recession and erythematous gingiva on the periodontal tissues.

### 3.1. Categorization of the Complications according to Their Acute or Chronic Nature

In a review paper by Campbell et al., the sequelae of piercing were categorized into acute and chronic [14] and the postoperative complications of oral piercing were analyzed. Trauma of the mucosa may include immediate responses for example, pain, swelling, hemorrhage, and local infection [10, 15] or postoperative complications including dysphonia, dysphagia, problems with mastication, and the occurrence of galvanic currents [6, 16] as well as contact dermatitis [17]. However, the aforementioned defects have not been proved to be deleterious to the tissues.

Pain has been reported as the most common consequence of oral piercing and the most common cause for the patients to seek consultation (52% of the examined cases) [18]. López-Jornet et al. have indicated that the mean pain intensity score based on a 0–10 scale visual analog scale (VAS) is 4. Furthermore, it is mentioned that in only 6 percent of the cases did the patients present with granulation tissue around the piercing and 20 percent exhibited increased levels of salivary flow. They also mention that harm to the ear (perichondritis and deformity) has been observed but not in their case report [18].

Some of the chronic consequences may involve postoperative hemorrhage and hyperplastic tissue. Vessels and vascular nerves may be cut during piercing procedure [19]. Significant absence of blood may lead to hypotensive collapse [20]. It is stated that prolonged bleeding, hematomas, and disturbance to the healing of the injuries are consequences of oral piercings [16].

Other chronic postoperative outcomes may include widening of the piercing hole [10], chemical burns related to excessive aftercare [21], paresthesia [15] sialadenitis [22], lymphadenitis, [23–25], sarcoid-like formations [26], granulomas, and scar tissue formation [16, 27]. Short shanks may result in overgrown tissues [28], whereas long shanks may result in hyperplastic tissue reaction and the presence of plaque and tartar [9, 10, 16, 29].

Intraoral piercings seem to be the culprit for the formation of hypertrophic keloid tissue, characterized by the production of an interstitial mucinous material on the collagen of connective tissue [29, 30]. Streptococcal pharyngitis, unpleasant itching sensation, and eczematous skin rash have also been reported as systemic complications [9].

### 3.2. Categorization of the Consequences according to the Nature of the Tissue Involved

#### 3.2.1. Consequences to the Hard Tissues

Damage to the hard tissues of the mouth has been suggested.

In 1997, DiAngelis was the first to state that tongue piercings contribute to abrasion resulting in cold sensitivity at the lower first molar teeth as a result of the cracked-tooth syndrome [31]. Tongue jewellery, habitual biting or chewing of the device, barbell stem length, the size of the ornament attached to the barbell, and the type of material used in it may all cause trauma to the teeth [32]. This trauma may involve the enamel, the dentin, or even the pulp [6, 33, 34].

Moreover, four cases have been reported that showed fracture of some cusps of the teeth [6]. In a tongue piercing case report of German soldiers who were only included in the clinical examination, it has been cited that the pierced group exhibited more carious teeth than the nonpierced group (*P* < 0.001), more enamel fissures (*P* < 0.01), more enamel cracks (*P* < 0.001), and more recessions especially at the lingual surfaces of mandibular anterior teeth (*P* < 0.001) [12]. Opposed to this important difference is the ratio of groove-shaped abrasions that is almost the same in both groups [35].

It is underlined that excessive playing with the piercing may cause misaligned teeth and diastema [36].

#### 3.2.2. Consequences to the Soft Tissues

Damage to the soft tissues has been presented as well.

The most prominent aftermath of piercing is gingival recession that is measured by using Miller's classification of marginal tissue recession [37]. Gingival recession is usual on the labial aspect of the lower central incisors [6, 14, 23, 27, 38–40] and on the lingual aspect of mandibular central incisors [6, 14, 16, 22, 39, 41]. Campbell et al. have pointed out that gingival recession of the lingual side occurs after 2 years of piercing insertion [14, 39].

In addition, Brooks et al. state that logistic regression modeling indicated that age was a significant predictor of the prevalence of lingual recession with the possibilities of presenting lingual recession increasing by 1.17 for each year older than 14. Furthermore, Poisson regression indicated that age was the most important foreteller of the number of lingual sites with regression. The most common form of recessions is as a narrow, cleft-like defect on the lingual and buccal sides of the mandibular incisors [39], with depths of recessions of 2-3 mm or more frequently extending to or beyond the level of the mucogingival junction. Even when the recession is short, serious attachment loss may still occur [14]. Kieser et al. provide a table that consists of the numbers and percentages that indicate the proportions of gingival recession and abnormal tooth wear by site of piercing and type of recession. This table suggests that the majority of people with lip piercing had at least one labial part with gingival recession, whereas 33% of people with tongue piercing showed at least one lingual site with gingival recession. All the people with lip and tongue piercing presented with at least one part with gingival recession and their average number of recession increased. No important discrepancies were found regarding abnormal tooth wear and piercing type. It has been reported that the clinical picture of the tissues near the piercings was excellent in 66% of the cases. Three of the eight students showed trivial alterations in soft or hard tissues: chipping of four premolars (three on the right side and one on the left side), gingival recession of the labial side of lower central incisors, scar on the skin from the removed labrette in the midline of lower lip, and irritation of the skin around the ring in lower lip.

Moreover, irritation of the skin around the opening of the mouth has been observed along with redness and light swelling, caused either by saliva flowing or contact allergy [6, 19, 20, 42]. Inchingolo has grouped the complications to immediate and delayed ones. Some effects after piercing include persistent mucosal atrophy, erythematous palatal mucosa, transient alteration in taste, and leakage of blood and serum [9]. In a case report Antoszewski et al. have detected a lip piercing that had caused decubitus and necrosis of the mucous membrane. The explanation to this finding was that the mucous membrane is more prone than the skin to mechanical injuries. Necrosis occurred at the place of oral vestibule and brought about embedding of the stud into the tissues of the lip [43].

### 3.3. Categorization of the Complications according to Their Systemic Severity

#### 3.3.1. Local Infections

Farah et al. have categorized the infections caused by oral piercing into local and systemic when detailing the infections. They have also stated that it is the most frequent consequence of oral piercing [15, 44]. Local infections may be attributed to the accumulation of dental plaque and calculus at the sites of piercing [45].

#### 3.3.2. Systemic Infections

Systemic infections, on the other hand, are caused by microorganisms (which are common in the oral cavity) that enter the systemic blood circulation and this could prove to be detrimental to immunocompromized people [46]. Lick et al. have mentioned some contributing factors to infective endocarditis. They have cited rheumatic heart disease, congenital deformities, hypertrophic cardiomyopathy, mitral valve prolapse associated with murmur, and mitral calcification [46–49]. Some life-risk situations have been reported including the development of cerebral brain abscesses [50] and Ludwig's angina [51]. López-Jornet et al. in their case series and review of oral and facial piercings underline the possibility of infection by infectious diseases such as Hepatitis B, C, D, and G, possibly HIV infection, tetanus, and tuberculosis [18]. It was estimated that the time a piercing is worn is relative to the periopathogenic strength of the oral flora that inhabits the tongue piercings [12]. Aggregatibacter actinomycetemcomitans has been frequently detected.

### 3.4. Consequences to the Oral Health according to the DMF Index

Regarding the DMF index, no statistically important discrepancies were evident between the groups about dental health [52]. However, it is stated that the number of students in this particular study of Campbell et al. was small and thus the statistical differences cannot easily be detected [14]. This specific study was the first to count salivary flows and compare them with a control group. More specifically, increased rates of flow were found in 63% of the students in comparison to 26% of the controls.

## 4. Awareness of the General Population Regarding the Sequelae of Tongue Piercing and of the Means of Reducing These Consequences

It has been pointed out in a plethora of articles that a statistically great proportion of the population which has undergone the procedure is not aware neither of the potential drawbacks of piercing nor of the possible ways of handling the possible problems that may arise from piercing. Levin et al. in their study state that 225 (57,8%) of the participants in this study were clueless of the drawbacks of having an oral piercing [53]. According to Antoszewski et al., some authors have suggested that the staff that performs piercings should be better informed in order to perform better practice [54–57]. This could mean that the staff shall pay meticulous attention in taking a sufficient medical history of the patient (specific allergies, life threatening systematic diseases) [58] or it could mean more careful usage of sterilization techniques [59]. Ventä et al. also suggest that patients do not get any piercing when not inebriated or during festivals [52]. On the contrary, Kleser et al. cite that the negative consequences of oral piercing did not differ between the patients who had undergone the procedure by a professional and the patients who had undergone the procedure by an amateur [60]. Inchingolo et al. on their short research paper have outlined some techniques that have been proved to ameliorate or even diminish the consequences of oral piercing [9]. These techniques include the following:

- a cold liquid diet for the first day and then a soft food diet,
- applying ice externally for thirty minutes in the meantime of 45 minutes for 5 times a day,
- mouthwashing with 0,12% chlorhexidine after the first day for 5 times a day for the first ten days,
- decreasing the consumption of alcohol, cigarettes and caffeine as these substances interfere with the epithelial reconstruction as well as decreasing the chewing of tobacco and gum,
- replacement of the initial jewelry with a smaller one,
- aphonia is preferable as much as possible,
- paying meticulous attention to oral hygiene, especially at the site of piercing and during the period of healing,
- checking the status of the oral and perioral piercing as often as possible for the avoidance of infections [61–63].

According to Levin et al., more techniques could be mentioned besides those that are mentioned above, which include the removal of piercing when inflammation is present, the application of local debridement of tissues, the use of antibiotics, and the application of chlorhexidine mouth rinse [53].

## 5. Conclusion

Overall, oral piercing is a trend that has emerged again in the last decade in the West and it is evident mostly among young members of society. Many etiological factors have been suggested in the scientific literature which conduce a potential piercee to the decision of wearing oral piercing. Nevertheless, patients are not quite satisfactorily informed about the drawbacks and plausible consequences of oral piercing. In addition, practitioners are not satisfactorily informed either, as they do not use decontamination techniques nor do they receive enough scientific background regarding piercing techniques. Dentists should be informed as well and should be more prepared to recognize such patients, their oral situation, and ways of preventing the consequences deriving from oral piercings. Oral piercing is not harmless at all. In fact, the vast majority of the reviews and case reports that have been published concerning this issue have agreed that oral piercings pose both a hazardous direct and indirect risk to the soft and hard oral and perioral tissues and they may even pose life-threatening risks. The connection between tongue piercing and periodontal problems has been proved to be evident (mostly because the barbell acts as a harbor for dental biofilm) and it is without a doubt a challenge for dentists to prevent these negative consequences and to inform and to prevent patients from acquiring such piercings because reconstructive and regenerative techniques are not always successful. It will definitely be a challenge not only for the dental but also for the medical scientific community in the near future.
